# Effect of pH, Temperature, and Salinity Levels on Heavy Metal Fraction in Lake Sediments

**DOI:** 10.3390/toxics12070494

**Published:** 2024-07-05

**Authors:** Shengnan Zhao, Yunxi Zhao, Zhimou Cui, Hui Zhang, Jinda Zhang

**Affiliations:** 1 Water Conservancy and Civil Engineering College, Inner Mongolia Agricultural University, Hohhot 010018, China; zhaoyunxi@emails.imau.edu.cn (Y.Z.); czm941019@petalmail.com (Z.C.); 1758065306@emails.imau.edu.cn (H.Z.); 1105872218@emails.imau.edu.cn (J.Z.); 2 Inner Mongolia Water Resource Protection and Utilization Key Laboratory, Hohhot 010018, China; 3 State Gauge and Research Station of Wetland Ecosystem, Wuliangsuhai Lake, Inner Mongolia, Bayan Nur 014404, China

**Keywords:** Wuliangsuhai Lake, heavy metals, copper, zinc, fraction analysis

## Abstract

Heavy metals (HMs) in aquatic environments are characterized by high toxicity, a propensity for bioaccumulation, and non-degradability, and pose significant risks to biological communities. Previous studies of HMs in lakes have shown that the physical and chemical characteristics of the lake water may control both the migration of HMs in the sediments and the concentration of heavy metals in the lake water. In fact, the change in aquatic environments changes the heavy metal fraction in the sediment, which controls the release of HMs. In this paper, we investigated the effects of the pH, temperature, and salinity levels of overlying water on the chemical fraction of Cu and Zn in Wuliangsuhai Lake surface sediments. The results show that lower water pH and higher water salinity and temperature could increase Cu and Zn release from the sediment. An increase in pH led to changes in the speciation of solid fractions of Zn, namely increases in the residual fraction and decreases in the organic matter and sulfide, whereas acid-extractable and Fe-Mn oxide fractions remained largely the same. Increases in temperature and salinity led to opposite changes in the speciation of solid fractions, namely decreases in the residual fraction and increases in the organic matter and sulfide and Fe-Mn oxide fractions, whereas acid-extractable fractions remained largely the same. The effect of pH, temperature, and salinity on Cu fractions in the solids was much smaller. According to the ratio of the secondary phase to the primary phase (RSP), acidic, high-temperature, and high-salt conditions increase the release risks of Zn. Changes in water temperature have the greatest influence on the risk of Zn and Cu release from sediments, followed by the influence of salinity changes.

## 1. Introduction

Heavy metal (HM) pollution has attracted much attention due to the strong toxicity, strong bio-enrichment, persistence, and non-degradation of heavy metals which affect the health of aquatic ecosystems [1,2]. Copper (Cu) and zinc (Zn) pose a serious risk to the environment and human health due to their short- and long-term toxicity. As a common component of algicides and herbicides, copper has toxic effects on bacteria, plants, fish, and benthic invertebrates [3,4,5,6]. Zinc is an essential element for the survival of organisms, but when the concentration exceeds the critical level, zinc also has a certain toxicity, causing oxidative damage to aquatic organisms and inhibiting protease activity [7,8,9]. 

In water environments, HMs are more likely to be adsorbed and accumulated by sediments [10,11,12,13]. Lake sediments can serve as potential secondary sources of HMs, and may pose a long-term threat to aquatic ecosystems [14,15]. The transport and transformation of HMs in sediment are influenced by various environmental factors, such as pH, temperature, the concentration of dissolved oxygen, nitrogen content, phosphorus content, salinity, and bioturbation [16,17]. Elevated hypoxia, salinity, or nitrogen enhance metal release from sediments. Temperature can not only impact the reaction of metal residues in sediments, but also change the DO concentration and the dissolution of carbonates and hydroxides, thus influencing the release of metals [18,19]. In fact, the (im)mobility and thus toxicity of HMs in sediments depend on the fraction [10,20]. HM fractions in sediments are divided into acid-extractable, Fe-Mn oxide-bound, organic and sulfide-bound, and residual [21,22,23,24,25]. These different HM fractions may show large variations under the influence of varying environmental conditions [26,27,28,29]. The hot topics of research on the transformation of heavy metal fractions are concentrated on sludge treatment, while research on aquatic sediments is relatively scarce. The effects of Fulvic Acid (FA) on the adsorption and fraction distribution of heavy metals (HMs) in sediments showed that FA additions showed significant negative and positive correlations with the percentages of metals bound to carbonates and organic matter, respectively [30]. HMs in sediments are continuously transformed between different phases when the pH, temperature, dissolved oxygen, and salinity of water change. Unfortunately, the existing studies do not explain the phenomenon of the fraction changes of heavy metals in sediments. Although many authors have studied the fractions of heavy metals in sediments, the transformation of heavy metal fractions during release has not been deeply analyzed. The effect of heavy metal fractions upon release remains unclear, especially for Zn and Cu-polluted lake sediment. 

Nearly half of the largest lakes in the world (surface area > 500 km^2^) are located above 40° N parallel [17] and are seasonally ice-covered. Fifty million lakes around the world freeze every year [31]. Compared with subtropical and tropical lakes, seasonal ice-covered lakes have certain particularities: The seasonal variation in dissolved oxygen in water is large [32]. The reoxygenation process formed by natural aeration changes greatly due to the ice-sheet barrier during the ice-covered period. In particular, when snowfall occurs during the ice-capped period, the content of dissolved oxygen will be zero (extreme anaerobic conditions) [33,34,35,36,37]. In seasonally frozen lakes, the presence of ice sheets changes light transmission into the water and affects the light-field environment of subglacial water. Photosynthesis and respiration in the lake are affected, which affects the pH of the lake water [38,39,40,41]. The existence of ice causes seasonal differences in the annual heat budget of Wuliangsuhai Lake, and the lake water temperature changes dramatically [32]. The temperature of the water is lower (0.1–1.5 °C) in the ice-covered period, but increases to 20–30 °C in the ice-free period [32]. During the growth of lake ice, solutes migrate from the ice to the water, increasing the concentration of solutes in the water [38]. Obviously, the seasonal lake water environment varies greatly during the year, and the environmental changes will affect the process of releasing HMs from sediments. In contemplating the mechanisms of HM release from sediments, we are faced with admitting that we lack basic knowledge for seasonally ice-covered lakes. Therefore, considering the characteristics of annual water environment changes in seasonal ice-covered lakes, it is necessary to analyze the fraction changes of heavy metals in sediments before and after their release under the influence of pH, temperature, and salinity.

In this study, Wuliangsuhai Lake, a shallow lake in China, was used to study the effect of pH, temperature, and salinity levels on heavy metal fractions. The main objectives of this study were to (1) investigate the different binding forms of Cu and Zn onto sediments at nine representative sediment sites in the lake in the freezing period; (2) analyze the changes in HM fractions in sediments before and after the release of HMs. The effects of different temperatures, pH, and salinity levels on the migration transformation and environmental risks of heavy metals (Cu and Zn) in sediments were investigated by analyzing the changes in the heavy metal content and fraction.

## 2. Materials and Methods

### 2.1. Study Site

Wuliangsuhai Lake (40°36′~41°03′ N, 108°43′~108°57′ E) is in Inner Mongolia. The water area is 293 km^2^, with an average lake elevation of 1018.5 m, and the water depth is 1.1~2.77 m, with an average depth of 1.78 m. The lake is the largest lake wetland in the Yellow River basin and is an extremely rare large multifunctional lake in the global arid grassland and desert regions. The lake is frozen from November to March, with the ice thickness ranging from 0.3 to 0.6 m. The wind speed is between 2 and 4 m/s, the annual sunshine duration is 2900–3200 h, the average annual temperature is 5.6–7.4 °C, the average annual precipitation is 224.2 mm, and the water surface evaporation is 2000–2600 mm. A large amount of agricultural drainage, industrial wastewater, and domestic sewage flow into Wuliangsuhai Lake every year. The annual average total phosphorus (TP), total nitrogen (TN), Chlorophyll-a (Chl.a), transparency (SD), and dissolved oxygen (DO) were 0.05 mg/L, 1.41 mg/L, 9.04 μg/L, 1.19 m, and 10.7 mg/L, respectively, in 2020. The trophic level index was 49.9 (mesotrophic) in 2020 [42]. Some areas of the lake are heavily polluted by heavy metals and the main polluting elements are Cd, Cu, and Hg [43].

### 2.2. Sampling Sites and Dates

**Figure 1:**
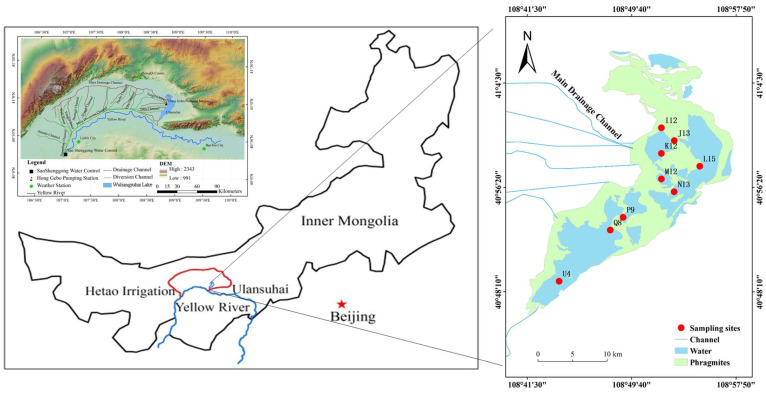
Location of different sampling sites of Wuliangsuhai Lake, Inner Mongolia, China.

Nine sampling sites were set in Wuliangsuhai Lake and located by GPS. Water and surface sediment (0–10 cm) samples were taken in January 2019 by using a column sampler (Figure 1). All of the surface sediment samples were placed into airtight polyethylene sample bags and then brought back to the laboratory for analysis. 

### 2.3. Laboratory Methods

(a) Experimental setup

Different experiments were conducted to analyze the release of Cu and Zn from the sediment samples. In the experiment, we used sediment samples from I12 (Figure 1). For each test, 1.0000 ± 0.0005 g (dry weight) of sediment was added into a 50mL conical flask, and then, 20 mL of deionized water was added. The conical flask was shaken at 200 r/min for 24 h. Then, the products were centrifuged at 4000 r/min for 20 min and filtered through a 0.45 μm cellulose acetate membrane. The sediment was sampled at the start and end of the experiment to determine the total amount and fraction of Cu and Zn. The centrifuge model used was Cence TDZ4-WS.

Three series of experiments were conducted: temperature, pH, and salinity in overlay water, respectively. According to the long-term monitoring data from 2011 to 2020, the water temperature of Wuliangsuhai ranges from 0.1 to 29.8 °C, the pH value of the water ranges from 7.62 to 9.25, the water’s electrical conductivity ranges from 1.12 to 6.2 mS/cm, and the TDS ranges from 0.96 to 8.82 g/L [42,44]. Therefore, we conducted the following tests:

(1) Maintain the salinity and pH value of the water, and change the water temperature. The temperature levels of the water in the controlled group were 5 °C, 15 °C, 25 °C, and 35 °C. The pH value of the water was 7, and the salinity was 1.5 g/L.
(2) Maintain the temperature and salinity of the water, and change the pH value of the water. The levels of pH in the water were 5, 6, 7, 8, and 9. The temperature of the water was 25 °C, and the salinity was 1.5 g/L.
(3) Maintain the temperature and pH value of the water, and change the salinity of the water. The levels of salinity in the controlled group were 0.5 g/L, 1.5 g/L, 2.5 g/L, 3.5 g/L, 4.5 g/L, and 5.0 g/L. The pH value of the water was 7, and the temperature was 25 °C.

Each experiment was set up with three parallel samples.

(b) Analysis of Samples
  (b-1) Physical characterization

The organic matter, pH, Eh, and particle size in the sediments were analyzed. 

Organic matter (LOI%): (1) First bake the prepared crucible at 105 °C for 1 h, take it out and cool it to room temperature in the dryer, and record the mass as m_0_. (2) Weigh 3~5 g of the sediment sample through a 100-mesh sieve into the crucible, bake it at 105 °C for 1 h, cool it in the dryer to room temperature after taking it out, and record the mass as m_1_. (3) Put the sediment sample and the crucible into the Muffle furnace at 550 °C for 2 h, and then put them into the dryer to cool to room temperature, and record the mass as m_2_. LOI% = ((m_1_ − m_2_)/(m_1_ − m_0_)) × 100%.

pH: Weigh 10 g of the air-dried sample with a balance of 1/10,000 g accuracy (0.0001 g), and then take a 50 mL beaker, sieve the sample with a 2 mm aperture and place it in it, add 25 mL of water to remove carbon dioxide (sediment–water ratio: 1:2.5), stir it with a glass rod for 1 min, and then measure the pH after letting it stand for 30 min.

Particle size: Put 0.35 g of the sample into a 100 mL beaker, and then process it according to the following steps. (1) Add 10 mL of 10% H_2_O_2_ to remove the organic matter and heat it on the heating plate until the reaction is calm. (2) Add 10 mL of 10% HCl and remove the carbonate after the reaction is complete. (3) Add distilled water to the beaker and let it stand for 24 h. (4) Extract the supernatant, add 10 mL of(NaPO_3_)_6_ dispersant with a concentration of 0.05 mol/L, place it for 2~3 min in the ultrasonic shaker to make it fully dispersed, and then test it on the laser particle size analyzer (BT-9300ST, Dandong Bettersize Instruments Ltd., Dandong, China).

(b-2) The total Cu and Zn

The total amount of Cu and Zn in the sediments was determined according to the following procedure: Place 0.3~0.4 g of the sample in a PTFE crucible, add 5 mL of premium pure hydrochloric acid, and heat it on an electric heating plate (130 °C). When the sample is digested to about 2~3 mL, take it off and cool it. Then, add 10 mL of (HClO_4_/HNO_3_ = 1:4), 5 mL of hydrofluoric acid, and 10 mL of superior pure nitric acid, move it to an electric heating plate, cover it, and heat it to decompose the sample until you have about 2 mL of liquid left. Add a little ultrapure water until the material in the crucible is viscous and move the liquid in the crucible into the 25 mL test tube to be measured; the volume is fixed to 25 mL.

(b-3) Cu and Zn fraction

Extract the fraction according to the European Community Bureau of BCR sequential extraction procedure [45], in which HMs in sediments are divided into acid-extractable, Fe-Mn oxide-combined, organic matter and sulfide-combined, and residual fractions. The experimental process is presented in Table 1.

### 2.4. Quality Assurance and Quality Control

Quality control was carried out by using a blank sample, blind sample, and standard sample. Three replicates were performed during the measurement. The quality control analysis revealed that the measurement error was less than 5%. The recoveries were 95~110%. All of the glassware were soaked for 48 h in 2 mol∙L^−1^ high-purity nitric acid and later washed in ultrapure water. All samples were quality-controlled according to GBW07305a.

### 2.5. Data Processing and Statistical Analysis

Different fractions of HMs have different bioavailability and geochemical characteristics, among which the acid-extractable, Fe-Mn oxide-combined, and organic matter and sulfide fraction can be used biologically, known as the secondary phase, while the residue fraction is not bioavailable and is regarded as the primary phase. The distribution ratio of HMs in the primary and secondary phases can reflect whether the sediments are polluted and the characteristics of pollution degree to a certain extent. Therefore, the RSP is proposed to evaluate the pollution degree of HMs in sediments from Wuliangsuhai Lake [45].

RSP = Msec/Mprim

where RSP is the ratio of the secondary phase to the primary phase; Msec is the secondary phase. Msec is the sum of the acid-extractable, Fe-Mn oxide-combined, and organic matter and sulfide-combined phases. Mprim is the primary phase, which is the primary mineral in the sediment, namely the residual HMs. The degree of heavy metal pollution can be divided into four grades [46]: RSP ≤ 1 (uncontaminated); 1 < RSP ≤ 2 (slight pollution); 2 < RSP ≤ 3 (moderate pollution); RSP > 3 (severe pollution).

Statistical Analysis: The statistical tests and analysis for this study were conducted using SPSS 20.0.0. Figures were generated using Origin 9.0 and excel 2016.

## 3. Results and Discussion

### 3.1. Sediment Properties

The sediment particles smaller than 4 μm were 4.14~24.93%, those of 4~64 μm were 23.91~69.17%, and those greater than 64 μm were 16.03~71.35%. The grain size distribution of sediments in Wuliangsuhai Lake was mainly silt, which was maintained at the level of medium silt to fine sand. The fluctuation range of the pH value of the sediments was always between 7.6 and 9.6, indicating that the sediments were alkaline. The average content of organic matter in the sediments was 9.9%. The conductivity of the sediments varied from 103.6 to 3960 μS/cm. The average EC value was 1341.5 μS/cm, indicating that the salinity of the lake sediments was high.

### 3.2. Total Amount and Fraction of HMs in Sediments

**Figure 2:**
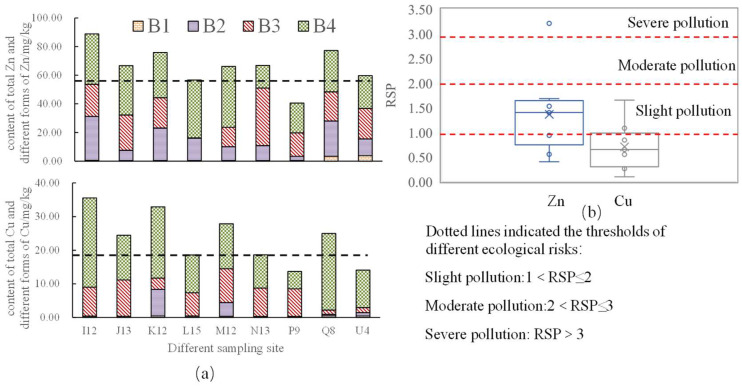
(**a**) The figure shows the total amount and chemical fraction distributions of Cu and Zn in the nine selected sediments of Wuliangsuhai Lake. 56.14 mg/kg was the reference levels of Zn [47]. 19.3 mg/kg was the reference levels of Cu [47]. The reference levels were local geochemical background values. (**b**) The figure shows the RSP of Cu and Zn in the nine selected sediments.

The Cu concentration varied from 13.6 to 35.6 mg/kg, with an average of 23.4 mg/kg. The Zn concentration varied from 40.6 to 88.8 mg/kg, with an average of 66.5 mg/kg (Figure 2). The regional geochemical background values were often employed as the reference values to probe the contamination level of the sediments. Thus, as shown in Figure 2a, Zn exhibited the highest contamination level where the metal concentration exceeded the reference values (denoted as the dotted line in the figure for 88.9% of the samples). In addition, the concentration of Cu of 55.6% of the samples was observed to exceed the reference levels.

Bioavailability and migration can be calculated by using various forms of HMs to analyze the potential risks of HMs. The acid-extractable fraction of Cu and Zn accounts for 1.8% and 1.6% of the total amount, respectively. The Fe-Mn oxide-combined fraction of Cu and Zn accounts for 5.3% and 21.4% of the total amount, respectively. The organic matter and sulfide-combined fraction of Cu and Zn accounts for 30.2% and 30.4% of the total amount, respectively. The residual fraction of Cu and Zn accounts for 62.5% and 46.6% of the total amount, respectively. The RSP was used to analyze the pollution risk of HMs. Thus, as shown in Figure 2b, the Zn concentration exceeded the severe pollution reference values for one sample. In addition, the concentration of Cu of two samples and of Zn of four samples was observed to exceed the slight pollution levels. The pollution of Zn was higher than that of Cu.

The content of the acid-extractable fraction at our nine sediment stations in Wuliangsuhai Lake sediment was low. Figure 2a shows that most Cu was in the residual fraction in most samples. Second, Cu also had a high organic matter and sulfide-combined fraction (B3), in line with many other sediment studies. Cu had a strong coordination ability with organic matter [48]. Wuliangsuhai Lake is a typical lake with high organic matter content [49]. The organic matter and sulfide-combined fraction of Zn was relatively high, but less than that of Cu. Zn in the nine sampling sites was mainly in the Fe-Mn oxide-combined fraction, which was mainly due to the high stability constants for these Zn-Fe-Mn oxide-combined phases.

The sum of the acid-extractable, Fe-Mn oxide-combined, and organic matter and sulfide fractions was high; Cu and Zn were 37.4% and 53.4%, respectively, which were sensitive to changes in water environment. This indicates that Cu and Zn would be released into water when environmental conditions change, resulting in water environment pollution.

### 3.3. Metal Release and Fraction Changes in Sediments Caused by pH, Temperature, and Salinity Changes

**Figure 3:**
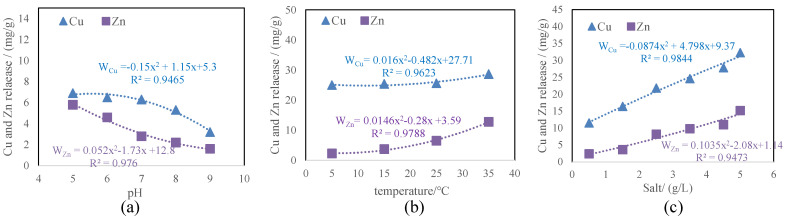
(**a**) The relationship between Cu and Zn release from the sediment and pH. (**b**) The relationship between Cu and Zn release from the sediment and temperature. (**c**) The relationship between Cu and Zn release from the sediment and salt content.

Figure 3 shows the release of Cu and Zn from sediments under various pH, temperature, and salinity levels. A downward tendency of Cu and Zn released from the sediments with the increase in pH is obviously observed, while a rising tendency of Cu and Zn released from the sediments with the increase in temperature and salinity is seen. Figure 3 shows that the released amount of Cu and Zn in sediments was negatively correlated with water pH and positively correlated with water temperature and salinity.

#### 3.3.1. Metal Release and Fraction Changes in Sediments Caused by pH

**Figure 4:**
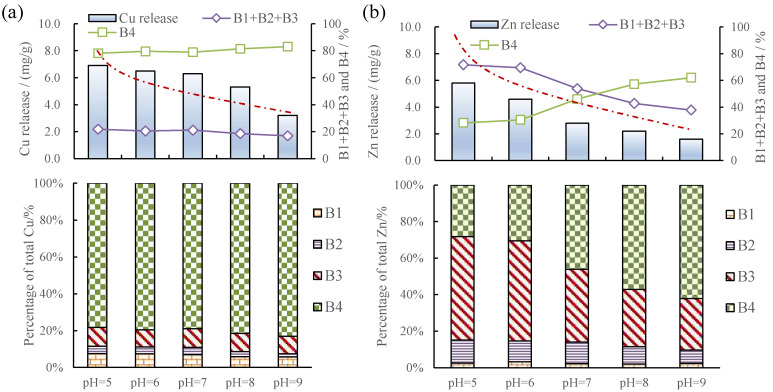
(**a**) Zn release and fraction changes in sediments due to pH changes. (**b**) Cu release and fraction changes in sediments due to pH changes; (B1 + B2 + B3) % indicates the sum of acid-extractable, Fe-Mn oxide-combined, and organic matter and sulfide-combined phases. Red dashed line indicates a trend.

Figure 4 shows that increases in pH led to increases in Zn adsorbed, as well as changes in the speciation of solid fractions, namely increases in the residual fraction and decreases in the organic matter and sulfide, whereas the acid-extractable and Fe-Mn oxide fractions remained largely the same. The effect of pH on Cu release was similar, but the effects on speciation in the solids was much smaller. By gradually increasing the pH in the water from around 4 to 9, Cu release decreased by 59% (Figure 4b) and the decrease in Zn release was 76% (Figure 4a). The proportion of the sum of the acid-extractable, Fe-Mn oxide-combined, and organic matter and sulfide-combined fractions of Zn and Cu decreased from 78.2% to 37.8 (Figure 4a) and from 23.8% to 16.9% (Figure 4b), respectively, with the increase in pH from 4 to 9. The proportion of the organic matter and sulfide-combined fraction of Zn decreased from 61.7% to 28.1% (Figure 4a). The change in pH mainly affected the organic matter and sulfide-combined fraction.

The capacity of particles to adsorb heavy metal ions increased with the increase in pH. The surface charge of clay minerals and organic matter increased in the sediment with increasing pH, the capacity of the sediment to adsorb HMs increased, and the passive adsorption of HMs was transformed into transition adsorption [50]. As pH decreases, H^+^ content increases, and more H^+^ competes for ligands with dissolved metals, thus promoting the release of metals [51]. The decrease in pH led to the dissolution of carbonate and hydroxide, and the competitive effect of H^+^ also increased the analytical amount of metal ions and obligated adsorption for ligand exchange, because both H^+^ and OH^−^ can react with the hydrated groups on the surface of metal-hydrated oxides. The resolution of the adsorbed anions was enhanced over a wide pH range. Therefore, the more alkaline the water, the less sediment release there is [52]. Organic matter and sulfide-combined fractions were mainly HMs bonding with organic matter such as animal and plant remains in sediments and with humus and sulfide mineral particles. The elevated acidity affected the release of sediment-associated metals, especially for the metals that have a high affinity for the organic matter and sulfide fraction. The decrease in pH and the increase in H^+^ concentration promote the dissolution of the residual state in the sediment and reduce the adsorption point. This increases the release of the sediment.

#### 3.3.2. Metal Release and Fraction Changes in Sediments Caused by Temperature

**Figure 5:**
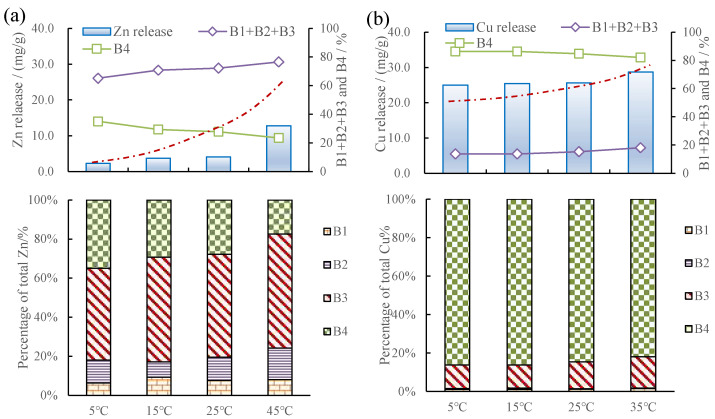
(**a**) Zn release and fraction changes in sediments due to temperature changes. (**b**) Cu release and fraction changes in sediments due to temperature changes.

An ascending tendency of Cu and Zn released from the sediments with the increase in temperature was obviously observed, but significant differences were observed for the release levels of the two sediment-bound metals. From 5 °C to 45 °C, the release of Cu increased by 55% (Figure 5b), while that of Zn increased by 783% (Figure 5a). Figure 5 shows that increases in temperature led to increases in Zn release, as well as changes in the speciation of solid fractions, namely decreases in the residual fraction and increases in the organic matter and sulfide fraction, whereas the acid-extractable and Fe-Mn oxide fractions remained largely the same. The effect of temperature on Cu release was similar, but the effects on speciation in the solids was much smaller. The fraction of Zn changed noticeably. In particular, when the temperature was 35 °C, the organic matter and sulfide-combined fraction of Zn in the sediments increased mostly from 47.0% at 5 °C to 55.8%, respectively. No matter how the temperature changed, the residual fraction was the main form of Cu which accounts for 80% of the total.

From a thermodynamic point of view, the adsorption process was exothermic as it occurred spontaneously. As the temperature rose, the ions were easily desorbed from particles, making the release rate increase. The organic matter and sulfide-combined fraction was also one of the carriers of adsorption, and the adsorption between organic matter and HMs is strong selective adsorption. Temperature changes can affect the adsorption processes. With the increase in temperature, the organic matter and sulfide-combined fraction increased, and the desorption capacity also increased, thus increasing the released amount from the sediment.

#### 3.3.3. Metal Release and fraction Changes in Sediments Caused by Salinity Changes

**Figure 6:**
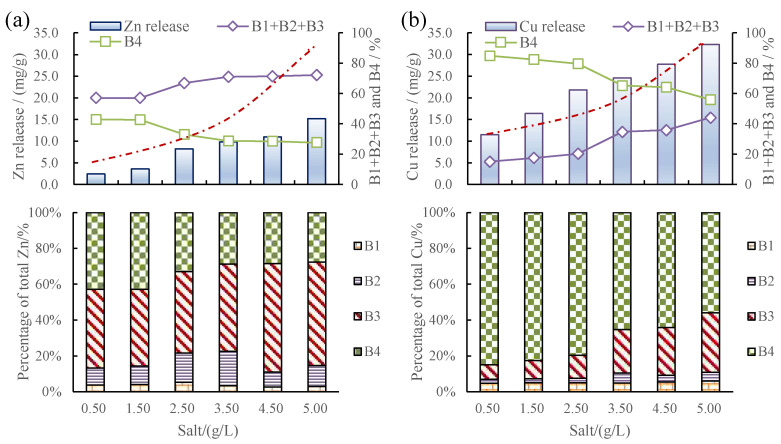
(**a**) Zn release and fraction changes in sediments due to salinity changes. (**b**) Cu release and fraction changes in sediments due to salinity changes.

Zn was thought to experience more salinity effects than Cu. The released amounts of Cu and Zn were 11.5 and 2.4 at a salinity of 0.5 g/L, respectively. The released amounts of Cu and Zn were 32.3 and 15.2 at a salinity of 5.0 g/L, respectively. Cu release increased by 180.8% and the increase in Zn release was 534%. Figure 6 shows that increases in salinity led to increases in Zn release, as well as changes in the speciation of solid fractions, namely decreases in the residual fraction and increases in the organic matter and sulfide fraction, whereas the acid-extractable and Fe-Mn oxide fractions remained largely the same. The effect of temperature on Cu release was similar. When the salinity was 5.0 g/L, the sum of the acid-extractable, Fe-Mn oxide-combined, and organic matter and sulfide-combined fractions of Zn and Cu in the sediments increased by 15.2 and 29.1%, respectively, compared to those at a salinity level of 0.5 g/L. In particular, when the salinity was 5.0 g/L, the organic matter and sulfide-combined fraction of Cu and Zn in the sediments increased mostly from 8.1% and 43.9% to 33.2% and 57.7%, respectively, demonstrating that salinity mainly affects the content of the organic matter and sulfide-combined fraction.

Both laboratory and field experiments indicate that with the increase in the salinity of water, the migration of HMs in sediments also increases. Lores and Pennock [53] suggested that complex interactions between salt ions, humic acids, and metals lead to fraction changes in Cu and Zn in sediments. In general, Cu and Zn in sediments were easily adsorbed by functional groups in organic matter. The higher the salinity, the more the solubility of organic matter will increase, thus enhancing the complexing ability of organic matter. Therefore, with the increase in salinity, the organic matter and sulfide fraction of HMs in sediments will significantly increase. In addition, humus decomposes to form a complex of humic acid and Cu^2+^ and Zn^2+^ in sediments, thus increasing the content of the Cu and Zn organic matter and sulfide-combined fraction.

#### 3.3.4. Relationships of Cu and Zn Fraction in Sediments with the Released Amounts

Linear regression was used to analyze the effect of Cu and Zn fractions on the released amount in sediments under changes in water environmental conditions, and the results are shown in Table 2. Under the effects of water pH, the release of Cu and Zn was positively correlated with the Fe-Mn oxide-combined fraction in the sediments. In addition, the R^2^ values of the regression analysis with the Fe-Mn oxide-combined fraction were 0.870 for Cu and 0.873 for Zn. Under the temperature control condition, the release of Cu and Zn in sediments was positively correlated with the concentration of organic matter and sulfide, and the R^2^ values were 0.941 and 0.778. For Zn, there were positive correlations between the released amount of Zn and the Fe-Mn oxide-combined fraction concentration in sediments, with an R^2^ value of 0.884. Under the salinity control condition, Zn and Cu release was positively correlated with the organic matter and sulfide-combined fraction in sediments, with R^2^ values of 0.948 and 0.823, respectively. In water pH-, temperature-, and salinity-controlled conditions, significantly negative correlations between the released amount of Cu and Zn and the residual fraction concentration in sediments were observed, with R^2^ above 0.95. With the increase in the released amount, more of the residual fraction was transformed into the organic matter and sulfide-combined fraction and the Fe-Mn oxide-combined fraction.

### 3.4. Implication of pH, Salinity, and Temperature Changes for HM Ecological Risk

**Figure 7:**
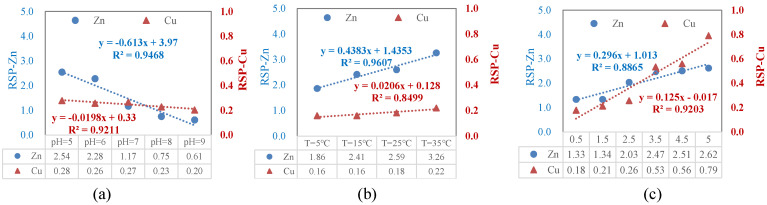
(**a**) The implication of pH changes for the metal ecological risk. (**b**) The implication of temperature changes for the metal ecological risk. (**c**) The implication of salinity changes for the metal ecological risk.

The risk changes of Cu and Zn under different treatment conditions were evaluated based on the RSP. Figure 7 shows the risk changes of Cu and Zn from sediments under various pH, temperature, and salinity stresses. When pH = 4, RSP_Zn_ = 3.60, which belongs to severe pollution with high risk; when the pH was 5–7, 2 < RSPZn < 3, which belongs to moderate pollution risk; when the pH was greater than 7, RSPZn was in a risk-free state (Figure 7a). The higher the temperature, the greater the risk of Zn migration. When the temperature was 5–15, 1 < RSP_Zn_ < 2, which indicates a risk of mild pollution. Temperatures above 25 °C were linked to a high risk of pollution (Figure 7b). The higher the salinity, the greater the risk of Zn migration. When the salinity was between 0.5 and 2.5 g/L, 1 < RSP_Zn_ < 2, which indicates a risk of slight pollution. At salinity levels higher than 2.5 g/L, 2 < RSP_Zn_ < 3, indicating a moderate pollution risk (Figure 7c). The risk variation trend of Cu in sediments under different pH, temperature, and salinity stresses was like that of Zn, but there was no risk because the RSP was less than 1.

It can be seen from Figure 7 that the varying gradients of different conditions have different impacts on the risk of HMs in sediments. In order to explore which factor has the greatest influence on the release risk, the released amounts in different environments were linearly fitted and derived to obtain Δy/Δx. For Zn, ΔRSP/ΔpH = −0.613, ΔRSP/ΔT = 0.483, and ΔRSP/ΔSalt = 0.296 (Figure 7). So, a gradient of water temperature has the greatest influence on the risk of heavy metal release in sediments, followed by the influence of salinity. The water temperature of the lake in the frozen period varies sharply, ranging from 0 to 4 in winter and reaching up to 30 in summer. This temperature gradient is large, which causes great risks to the release of the heavy metal Zn in the lake sediments. Increasing temperature enhances the migration risk of Zn and Cu. The salt in the lake ice migrates into the subglacial water and increases the salt content in the subglacial water. The salinity of Wuliangsuhai Lake water is 2.0 g/L in the glacial period and 1.5 g/L in the non-glacial period. The change in the salinity gradient will also affect the risk of heavy metal release; the addition of NaCl increased the exchangeable fraction (B1 + B2 + B3) of Cu and Zn, suggesting that increased water salinity induced by ice during the winter season may enhance their mobility.

## 4. Conclusions

Increases in pH led to increases in Zn release, as well as changes in the speciation of solid fractions, namely increases in the residual fraction and decreases in the organic matter and sulfide fraction, whereas the acid-extractable and Fe-Mn oxide fractions remained largely the same. Increases in temperature and salinity led to increases in Zn release, as well as changes in the speciation of solid fractions, namely decreases in the residual fraction and increases in the organic matter and sulfide and Fe-Mn oxide fractions, whereas the acid-extractable fractions remained largely the same. The effect of pH, temperature, and salinity on Cu release was similar, but the effects on speciation in the solids was much smaller.

The water temperature gradient was found to have the greatest influence on the risk of HM release from sediments in Wuliangsuhai Lake, followed by the influence of salinity.

## Figures and Tables

**Table 1:** The BCR sequential extraction procedure.

Step	Soil Phases	Extractant	Shaking Time and Temperature
B1	Acid-extractable	20 mL 0.11 mol/L CH_3_COOH	shaken at 25 °C for 30 r/min for 16 h and then centrifuged at 4000 r/min for 20 min
B2	Fe-Mn oxide-combined	20 mL 0.1 mol/L NH_2_OH·HCI (pH = 2)	shaken at 25 °C for 30 r/min for 16 h and centrifuged at 4000 r/min for 20 min
B3	Organic matter and sulfide-combined	5 mL 8.8 mol/L H_2_O_2_ (pH = 2) then 5 mL 8.8 mol/L H_2_O_2_, add 25 mL 1mol/L NH_4_OAc (pH = 2)	water bath at 25 °C for 1 h then 85 °C for 1 h shaken at 25 °C for 30 r/min for 16 h and centrifuged at 4000 r/min for 20 min
B4	Residual	10 mL (HClO_4_:HNO_3_ = 1:4)	heated on hot plate to dryness

**Table 2:** Correlations between metal fractions and metals release.

	pH					T					Salinity				
	B1	B2	B3	B4	Total	B1	B2	B3	B4	Total	B1	B2	B3	B4	Total
R-Cu	0.913 *	0.87 *	0.622	−0.925 **	−0.369	0.52	0.497	0.941 *	−0.987 **	−0.764	0.892 *	0.715	0.948 **	−0.951 **	−0.396
R-Zn	0.723	0.873 *	0.98 *	−0.98 **	0.478	0.073	0.884 *	0.778	−0.956 **	0.485	−0.5	0.167	0.823 **	−0.95 **	−0.414

* Correlation was significant at the 0.05 level. ** Correlation was significant at the 0.01 level. R-Cu: the released amount of Cu. R-Zn: the released amount of Zn.

## Data Availability

Data are contained within the article.
